# Autosomal Dominant Hypercalciuria in a Mouse Model Due to a Mutation of the Epithelial Calcium Channel, TRPV5

**DOI:** 10.1371/journal.pone.0055412

**Published:** 2013-01-30

**Authors:** Nellie Y. Loh, Liz Bentley, Henrik Dimke, Sjoerd Verkaart, Paolo Tammaro, Caroline M. Gorvin, Michael J. Stechman, Bushra N. Ahmad, Fadil M. Hannan, Sian E. Piret, Holly Evans, Ilaria Bellantuono, Tertius A. Hough, William D. Fraser, Joost G. J. Hoenderop, Frances M. Ashcroft, Steve D. M. Brown, René J. M. Bindels, Roger D. Cox, Rajesh V. Thakker

**Affiliations:** 1 Academic Endocrine Unit, Nuffield Department of Medicine, University of Oxford, Oxford Centre for Diabetes, Endocrinology and Metabolism (OCDEM), Churchill Hospital, Headington, Oxford, United Kingdom; 2 MRC Mammalian Genetics Unit and Mary Lyon Centre, Medical Research Council, Harwell, Oxfordshire, United Kingdom; 3 Department of Physiology, Nijmegen Centre for Molecular Life Sciences, Radboud University Nijmegen Medical Centre, Nijmegen, The Netherlands; 4 Department of Physiology, Anatomy and Genetics, University of Oxford, Oxford, United Kingdom; 5 Academic Unit of Bone Biology, University of Sheffield, The Medical School, Sheffield, United Kingdom; 6 Faculty of Medical and Health Sciences, University of East Anglia, Norwich Research Park, Norwich, United Kingdom; University of Tokyo, Japan

## Abstract

Hypercalciuria is a major cause of nephrolithiasis, and is a common and complex disorder involving genetic and environmental factors. Identification of genetic factors for monogenic forms of hypercalciuria is hampered by the limited availability of large families, and to facilitate such studies, we screened for hypercalciuria in mice from an *N*-ethyl-*N*-nitrosourea mutagenesis programme. We identified a mouse with autosomal dominant hypercalciuria (HCALC1). Linkage studies mapped the *Hcalc1* locus to a 11.94 Mb region on chromosome 6 containing the transient receptor potential cation channel, subfamily V, members 5 (Trpv5) and 6 (Trpv6) genes. DNA sequence analysis of coding regions, intron-exon boundaries and promoters of *Trpv5* and *Trpv6* identified a novel T to C transition in codon 682 of TRPV5, mutating a conserved serine to a proline (S682P). Compared to wild-type littermates, heterozygous (*Trpv5* ^682P/+^) and homozygous (*Trpv5* ^682P/682P^) mutant mice had hypercalciuria, polyuria, hyperphosphaturia and a more acidic urine, and ∼10% of males developed tubulointerstitial nephritis. *Trpv5* ^682P/682P^ mice also had normal plasma parathyroid hormone but increased 1,25-dihydroxyvitamin D_3_ concentrations without increased bone resorption, consistent with a renal defect for the hypercalciuria. Expression of the S682P mutation in human embryonic kidney cells revealed that TRPV5-S682P-expressing cells had a lower baseline intracellular calcium concentration than wild-type TRPV5-expressing cells, suggesting an altered calcium permeability. Immunohistological studies revealed a selective decrease in TRPV5-expression from the renal distal convoluted tubules of *Trpv5* ^682P/+^ and *Trpv5* ^682P/682P^ mice consistent with a trafficking defect. In addition, *Trpv5^682P/682P^* mice had a reduction in renal expression of the intracellular calcium-binding protein, calbindin-D_28K_, consistent with a specific defect in TRPV5-mediated renal calcium reabsorption. Thus, our findings indicate that the TRPV5 S682P mutant is functionally significant and study of HCALC1, a novel model for autosomal dominant hypercalciuria, may help further our understanding of renal calcium reabsorption and hypercalciuria.

## Introduction

Kidney stone disease (nephrolithiasis) affects 12% of men and 5% of women by the seventh decade of life and has a recurrence rate of ∼10% per annum [1]. Approximately 80% of kidney stones contain calcium as calcium oxalate and/or calcium phosphate, and hypercalciuria is the most common metabolic abnormality found in such calcium stone formers [1], [2]. The aetiology of hypercalciuria may involve absorptive, renal, or resorptive mechanisms, depending on the site of the primary defect, resulting in intestinal hyperabsorption, impaired renal tubular reabsorption, or increased bone resorption, respectively [3]. In addition, hypercalciuria and nephrolithiasis may have a genetic aetiology, as 35–65% of patients with hypercalciuric nephrolithiasis have affected family members [2]. Moreover, twin studies have estimated the heritability of nephrolithiasis and hypercalciuria as 56% [4] and 52%, [5] respectively, and both may occur as polygenic quantitative traits, or as monogenic traits inherited as autosomal dominant, autosomal recessive or X-linked disorders [2], [6], a situation that is similar to that for many common clinical disorders, e.g. hypertension and diabetes mellitus [7], [8]. However, it is important to note that the polygenic forms of these diseases including hypercalciuric nephrolithiasis are more common, whereas the familial monogenic forms are rare [9], and that the study of both forms has yielded important and novel insights of homeostatic mechanisms and their roles in disease processes. This is well illustrated by studies of the different forms of hypercalciuric nephrolithiasis. Thus, genome-wide association studies, aiming to reveal gene variants contributing to polygenic traits,in Icelandic and Dutch populations identified susceptibility risk variants in the claudin 14 (CLDN14) gene for hypercalciuric nephrolithiasis, [10] and a study of Swiss renal calcium stone formers has reported an association between an ancestral haplotype defined by the non-synonymous polymorphisms of the transient receptor potential cation channel, subfamily V, member 6 (TRPV6), which resulted in a gain-of-function and absorptive hypercalciuria [11]. In addition, studies of monogenic (i.e. familial) forms of hypercalciuric nephrolithiasis have identified: an association between the human soluble adenylyl cyclase and an autosomal dominant form of absorptive hypercalciuria [12], [13]; gain-of-function mutations of the calcium-sensing receptor in autosomal dominant hypocalcaemia with hypercalciuria [2], [14]; mutations of the sodium-phosphate co-transporter solute family 34 member 3 (SLC34A3), in an autosomal recessive form of hypophosphataemic rickets with hypercalciuria [2], [15]; mutations of the chloride/proton antiporter, CLC-5, in Dent's disease, an X-linked recessive form of hypercalciuric nephrolithiasis [16]; and mutations of the bumetanide-sensitive sodium-potassium-chloride cotransporter (NKCC2), the renal outer-medullary potassium channel (ROMK), and the voltage-gated chloride channel, CLC-Kb in autosomal recessive forms of Bartter's syndrome type I-III, respectively, which are associated with hypercalciuria [15]. These latter studies have been successful as large families with the disorder were available. However, such families with monogenic forms of hypercalciuric nephrolithiasis are frequently unavailable, as this form of the disorder is rare and because hypercalciuric nephrolithiasis is a late-onset disorder, and therefore at the time of presentation of a kidney stone the parents of a proband may be deceased and younger family members may not have developed any manifestations of the disorder [17]. To overcome these difficulties and facilitate the identification of genetic abnormalities causing hypercalciuric nephrolithiasis, we embarked on studies to establish mouse models generated using *N*-ethyl-*N*-nitrosourea (ENU), a chemical mutagen that causes point mutations by alkylation of nucleic acids leading to mispairing and subsequent single base substitutions during DNA replication [18]. ENU mouse mutants, which can be associated with loss-of-function, hypomorphic, hypermorphic or dominant-negative effects [18], have been successfully derived for metabolic and renal disorders including a mouse model with obesity and hyperinsulinaemia caused by a V145E substitution in the leptin gene [19], and a mouse model for renal failure due to a C277S substitution in aquaporin-11 [20]. We now report the identification of an ENU-induced mouse mutant model for autosomal dominant hypercalciuria, HCALC1, due to mutation of the transient receptor potential cation channel, subfamily V, member 5 (*Trpv5*) gene.

## Results

### Identification of HCALC1 mice and Trpv5 mutation

**Figure 1:**
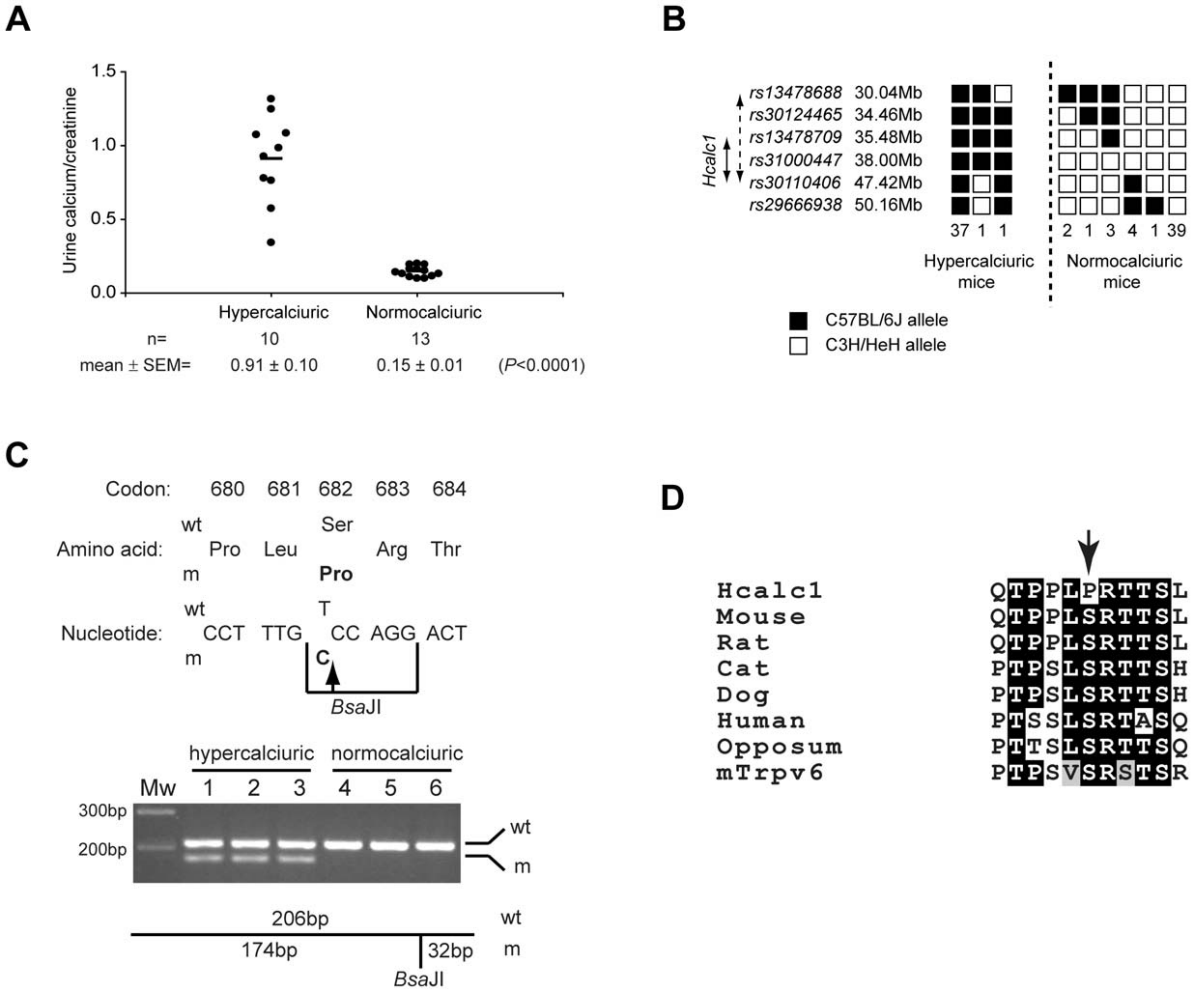
Hypercalciuria in HCALC1 ENU mutant mice and identification of a *Trpv5* mutation. (**A**) Urine calcium/creatinine ratios in 23 G2 offspring of the HCALC1 founder male revealed that 10 of the 23 mice were hypercalciuric, consistent with an autosomal dominant inheritance. Bar, mean calcium/creatinine values. (**B**) Haplotype analysis of 89 G2 mice (39 hypercalciuric and 50 normocalciuric) was initially undertaken separately in the hypercalciuric and normocalciuric mice, as the penetrance of HCALC1 was unknown. Haplotype analysis of the hypercalciuric mice localised *Hcalc1* to a 17.38 Mb interval on chromosome 6, flanked by *rs13478688* and *rs30110406* (broken double-headed arrow). Haplotype analysis using combined data for the hypercalciuric and normocalciuric mice identified the smaller interval, 11.94-Mb, flanked by *rs13478709* and *rs30110406* (solid double-headed arrow). The *Hcalc1* locus is inherited with the C57BL/6J haplotype from the F1 founder male. Filled box, C57BL/6J allele; and open box, C3H/HeH allele. Number of mice observed for each haplotype is shown beneath each column. (**C**) DNA sequence analysis of *Trpv5* identified a heterozygous T to C transition in codon 682 in hypercalciuric mice predicted to alter a wild-type serine (Ser) to a mutant proline (Pro). This mutation resulted in gain of a *Bsa*JI restriction enzyme site that was used to confirm the presence of the mutation in the 39 hypercalciuric mice (n = 3 shown) and its absence in the 50 normocalciuric mice (n = 3 shown). wt, wild-type; m, mutant. (**D**) Amino acid sequence alignment revealed evolutionary conservation of the wild-type mouse TRPV5 serine (S) residue at codon 682 (arrowed) in 5 species, as well as in mouse TRPV6 (mTrpv6). Identical residues are shaded black and conservative changes are shaded grey.

The HCALC1 founder mouse was identified from plasma and urinary biochemical analysis of F1 male offspring of ENU-mutagenised C57BL/6J male mice and wild-type C3H/HeH (C3H) female mice. The founder mouse, who was normocalcaemic, was found to have a urine calcium/creatinine ratio >10 SD above the mean of age-matched control males (2.49 *vs*. 0.26±0.20, respectively at age 16 weeks, 3.93 *vs*. 0.28±0.21, respectively at age 24 weeks), consistent with idiopathic hypercalciuria [21]._ENREF_17 The HCALC1 founder male was mated with normal C3H females and plasma and urinary analysis of the second-generation (G2) progeny revealed 10 of 23 offspring were normocalcaemic but had urine calcium/creatinine ratios >2 to 9-fold above the mean of age-matched control littermates. The occurrence of hypercalciuria in 43% of the progeny is consistent with an autosomal dominant phenotype (Figure 1A). The presence of hypercalciuria in the HCALC1 mice was not associated with nephrocalcinosis, as renal histology using von Kossa staining to detect calcium deposits, revealed that the frequency of interstitial renal cortical calcification (one or more calcified foci/renal cross-section) in HCALC1 and wild-type (unaffected) mice was similar (26% and 24%, respectively).

A genome-wide search using chromosome-specific single nucleotide polymorphisms (SNPs), at 20–30 cM intervals and DNA from 13 mice (10 hypercalciuric mice and 3 normocalciuric littermates) revealed co-segregation of the *Hcalc1* locus with chromosome 6 (LOD score  =  3.91, 0% recombination). Analysis using additional chromosome 6 SNPs in 89 G2 mice (39 hypercalciuric and 50 normocalciuric) demonstrated co-segregation of the *Hcalc1* locus with chromosome 6B1/B2, with a peak LOD score  =  26.8 at 0% recombination (Figure 1B). An analysis of the recombinants observed in the hypercalciuric mice revealed a 17.38 Mb interval flanked by *rs13478688* and *rs30110406*, and additional analysis including the normocalciuric mice indicated the critical interval containing the *Hcalc1* locus was between *rs13478709* and *rs30110406*, which is 11.94 Mb in size and contains 176 genes, including those for *Trpv5* and *Trpv6*.

Sequence analysis of the coding regions, intron-exon boundaries and promoter sequences of *Trpv5* and *Trpv6* [22], [23] using DNA from a hypercalciuric G2 mouse and wild-type C57BL/6J and C3H mice, did not identify a mutation in *Trpv6*. However, a heterozygous T to C transition in codon 682 of *Trpv5*, predicted to alter a wild-type serine (S) to a mutant proline (P), was identified in the hypercalciuric mouse (Figure 1C), resulting in gain of a *Bsa*JI restriction enzyme site, which was used to confirm the heterozygous mutation in all hypercalciuric mice, consistent with an autosomal dominant trait. The S682P mutation was absent in all normocalciuric mice (Figure 1C). The S682 residue, located in the cytoplasmic C-terminus of TRPV5, is evolutionarily conserved in the TRPV5 sequences of six species and also in TRPV6 (Figure 1D). Thus, the substitution of the polar serine residue for the nonpolar proline is likely to be a significant mutation and its effects on channel function were investigated.

### In vitro effects of TRPV5 mutation in HCALC1 mice

**Figure 2:**
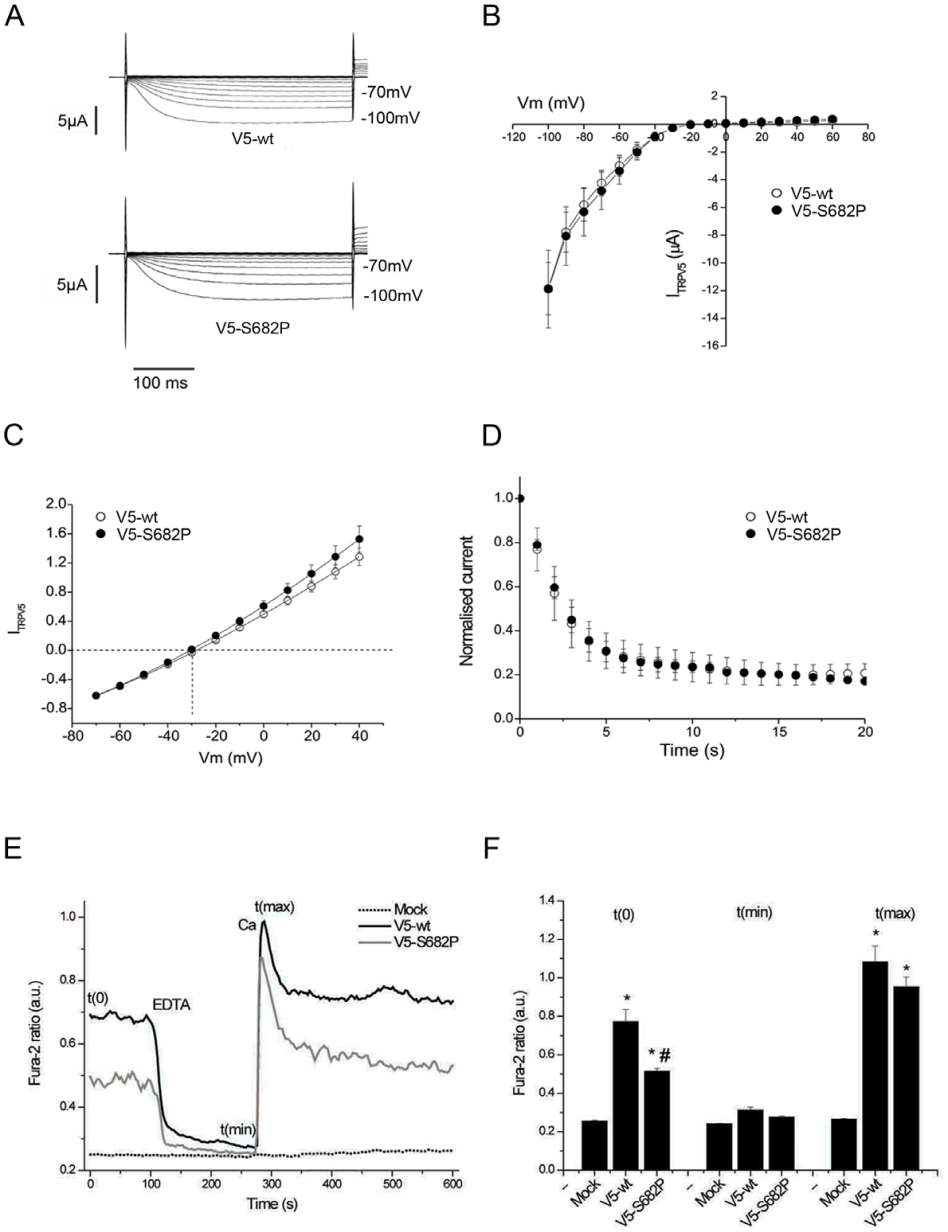
Channel characteristics of wild-type and mutant TRPV5. (**A**) Whole-cell currents in TRPV5-WT (V5-WT) and TRPV5-682P (V5-S682P) injected *Xenopus* oocytes recorded in response to 300 ms test pulses to various potentials (from −100 to +60 mV in 10 mV increments). Holding potential, 0 mV (N = 5). (**B**) Mean current-voltage relationships for TRPV5-WT and TRPV5-682P channels (N = 5). These current-voltage relationships are similar to those reported for TRPV5 channels [62]. (**C**) Mean whole-cell tail currents measured in TRPV5-WT and TRPV5-682P injected *Xenopus* oocytes during test potentials applied in 10 mV increments from −70 to +40 mV after a pre-pulse to −100 mV in TRPV5-WT and TRPV5-682P channels (N = 5). (**D**) Time-dependent inhibition of TRPV-WT and TRPV5-682P whole-cell currents. Oocytes were stimulated every 1 s. The peak current amplitude was normalised to that recorded during the first pulse (N = 4). (**E**) Representative trace of Fura-2 ratio in HEK293 cells transiently transfected with an empty EGFP vector (mock), or EGFP-tagged TRPV5-WT or TRPV5-S682P. Cells expressing EGFP were selected and monitored for changes in intracellular Ca^2+^ levels when extracellular Ca^2+^ concentrations were varied from 1.4 mM Ca^2+^ to 0 mM Ca^2+^ (2 mM EDTA) and 1.4 mM Ca^2+^ which was facilitated by superfusion. (**F**) Fura-2 levels under resting conditions (t0), minimal Fura-2 ratio after EDTA treatment (tmin) and peak level (tmax) upon administration of 1.4 mM Ca^2+^ after EDTA treatment. Average data of cells transfected with the empty vector (n = 7), TRPV5-wt (n = 24) and TRPV5-S682P (N = 24) from at least three independent experiments. * p<0.05 for TRPV5-WT or TRPV5-S682P versus cells transfected with empty vector. # p<0.05 for TRPV5-S682P versus TRPV5-wt transfected cells.

TRPV5 is an epithelial calcium channel which functions as a tetramer, is predominantly expressed in the renal distal convoluted tubule (DCT) and connecting tubule (CNT), and is involved in vitamin D-regulated renal calcium reabsorption [24], [25]. The effect of the mutation on TRPV5 channel characteristics was investigated by electrophysiological recordings in Xenopus oocytes (Figure 2A–D). These revealed no differences between wild-type and TRPV5-S682P channel properties, thereby indicating that either the S682P alteration was not a pathogenic mutation or that the S682P mutation may be having an effect through different mechanisms, such as impaired trafficking of the channel to the plasma membrane, which may not be detected in Xenopus oocytes; either because the ion channel protein is overexpressed in the heterologous expression system, or because the cellular machinery that regulates channel trafficking in Xenopus oocytes may differ from that of mammalian cells. We therefore assessed the effect of the mutation on TRPV5 channel characteristics in Human Embryonic Kidney (HEK)-293 cells. Similarly, to the Xenopus oocyte measurements, whole-cell patch clamp recordings in HEK293 cells did not reveal differences in current density carried by Na^+^ or Ca^2+^ between TRPV5-WT and TRPV5-S682P (Figure S1A–D). In addition, Ca^2+^ dependent inhibition of Na^+^currents was similar between TRPV5-WT and TRPV5-S682P (Figure S1E and F). The absence of any effect by the mutant may again be due to overexpression of ion channel proteins in the heterologous system. Moreover, it is important to note that the whole-cell patch clamp technique results in a dilution of the intracellular environment, and that this may reduce the effects of intracellular factors, which may be involved in channel trafficking. We therefore carried out further studies of the intracellular effects of the S682P mutation on calcium flux, using the calcium-responsive dye, Fura-2, as follows. HEK293 cells were transiently transfected with constructs encoding enhanced green fluorescent protein (EGFP)-tagged wild-type mouse TRPV5 (TRPV5-wt), mouse TRPV5 with the S682P mutation (TRPV5-S682P), or empty (mock) EGFP vector. EGFP positive cells were monitored for changes in intracellular calcium ([Ca^2+^]_i_) in response to extracellular calcium ([Ca^2+^]_o_) changes using the Ca^2+^-sensing dye, Fura-2 (Figure 2E). Transient expression of TRPV5-wt resulted in an elevated basal [Ca^2+^]_i_ level compared to mock-transfected cells, due to increased calcium permeability of the cell. When the cells were superfused with calcium-free medium, [Ca^2+^]_i_ in TRPV5-expressing cells decreased to levels similar to mock-transfected cells. Re-application of 1.4 mM Ca^2+^ solution induced a rapid increase of [Ca^2+^]_i_ followed by a gradual decrease back to basal levels (Figure 2E). No significant changes in [Ca^2+^]_i_ were observed in mock-transfected cells in response to Ca^2+^ depletion and Ca^2+^ re-application (Figure 2F). By comparison, cells transfected with TRPV5-S682P showed a lower basal [Ca^2+^]_i_ than TRPV5-wt-transfected cells (N = 24/group, p<0.001, Figure 2E–F). The TRPV5-S682P-transfected cells had a similar response in [Ca^2+^]_i_ to Ca^2+^ depletion and Ca^2+^ re-application as that observed in TRPV5-wt-transfected cells. These data suggest that the S682P mutation in TRPV5 affects baseline calcium permeability of the channel rather than calcium-induced channel inactivation, a feature consistent with the hypercalciuric phenotype observed in HCALC1 mice (Figure 1A). Renal excretion and histology were therefore further investigated in the HCALC1 mice.

### In vivo effects of TRPV5 mutation in HCALC1 mice

HCALC1 mice were interbred to generate wild-type (*Trpv5^+/+^*), heterozygous (*Trpv5* ^682P/+^) and homozygous mutant (*Trpv5* ^682P/682P^) mice. *Trpv5* ^682P/682P^ mice, were found to be viable, fertile and morphologically indistinguishable from their *Trpv5* ^+/+^ and *Trpv5* ^682P/+^ littermates. Mice between 12–20 weeks of age were housed in metabolic cages for 24 hours and urine samples collected for biochemical analysis [26]. *Trpv5* ^682P/682P^ mice were polydypsic and polyuric compared to *Trpv5* ^+/+^ mice (p<0.02, Table 1). In addition *Trpv5* ^682P/+^ and *Trpv5* ^682P/682P^ mice were hypercalciuric, hyperphosphaturic, and had an acidic urine (p<0.02, Table 1). Plasma calcium and phosphate concentrations were similar in *Trpv5* ^+/+^ and *Trpv5* ^682P/+^ mice, but *Trpv5* ^682P/682P^ male and female mice had significantly lower plasma calcium concentrations (p<0.02), whereas only the *Trpv5* ^682P/682P^ female mice were hypophosphataemic (p<0.02, Table 1). The lower plasma calcium concentrations in the *Trpv5* ^682P/682P^ mice were not observed to be associated with symptoms of neuromuscular irritability or seizures, presumably because they had mild hypocalcaemia. Indeed, plasma PTH concentrations in the *Trpv5* ^+/+^, *Trpv5* ^682P/+^ and *Trpv5* ^682P/682P^ mice were not significantly different (Table 1), but the plasma 1,25-dihydroxyvitamin D_3_ concentrations were significantly elevated in male and female *Trpv5* ^682P/682P^ mice (p<0.05 and p<0.02, respectively) (Table 1). The elevated circulating 1,25-dihydroxyvitamin D_3_ concentrations observed in the *Trpv5* ^682P/682P^ mice indicate that the mild asymptomatic hypocalcaemia, despite the severe renal loss of calcium in these mutant mice, is being maintained via a compensatory, 1,25-dihydroxyvitamin D_3_ mediated increase in intestinal calcium absorption, as reported in *Trpv5* ^-/-^ mice [25].

**Table 1:** Phenotypic characterisation of HCALC1 mice.

		Male			Female	
	wt	het	hom	wt	het	hom
Water intake (ml/24 hr)	3.22±0.18	3.90±0.24	5.70±0.47*^♯^	3.76±0.16	3.95±0.29	6.80±0.54*^♯^
Urine output (ml/24 hr)	1.22±0.08	1.53±0.14	2.26±0.30*	1.62±0.08	1.95±0.15	3.97±0.35*^♯^
Urine Ca^2+^/Cr	0.21±0.01	1.85±0.09*	5.09±0.37*^♯^	0.24±0.01	3.00±0.12*	6.57±0.40*^♯^
Urine Phos/Cr	11.7±0.5	14.4±0.4*	16.3±0.5*^♯^	10.1±0.5	12.3±0.6*	14.4±0.6*^♯^
Urine pH	6.92±0.07	6.43±0.06*	6.11±0.05*^♯^	6.75±0.05	6.14±0.07*	5.94±0.04*
Plasma calcium (mmol/l)	2.82±0.04	2.74±0.03	2.63±0.03*	2.77±0.04	2.80±0.04	2.62±0.04*^♯^
Plasma phosphate (mmol/l)	4.74±0.18	4.30±0.19	4.07±0.25	4.12±0.23	4.45±0.23	3.31±0.18*^♯^
Plasma PTH (pmol/l)	44.3±6.5	40.3±5.7	59.6±8.1	41.6±4.1	39.3±5.2	38.7±3.6
1,25 vitamin D3 (pmol/l)	46.5±12.7	77.2±6.0	181±45.9^$^	53.6±6.7	88.3±15.4	126.3±13.7*

Metabolic cage analysis of *Trpv5^+/+^* (wt), *Trpv5^682P/+^* (het) and *Trpv5^682P/682P^* (hom) mice for 24 hours (N = 15–72 mice/group). All data are presented as means±SEM. ^$^p<0.05 compared to *Trpv5^+/+^*, * p<0.02 compared to *Trpv5^+/+^*, ^#^p<0.02 compared to *Trpv5* ^682P/+^ mice, with Bonferroni correction for multiple comparisons.

**Figure 3:**
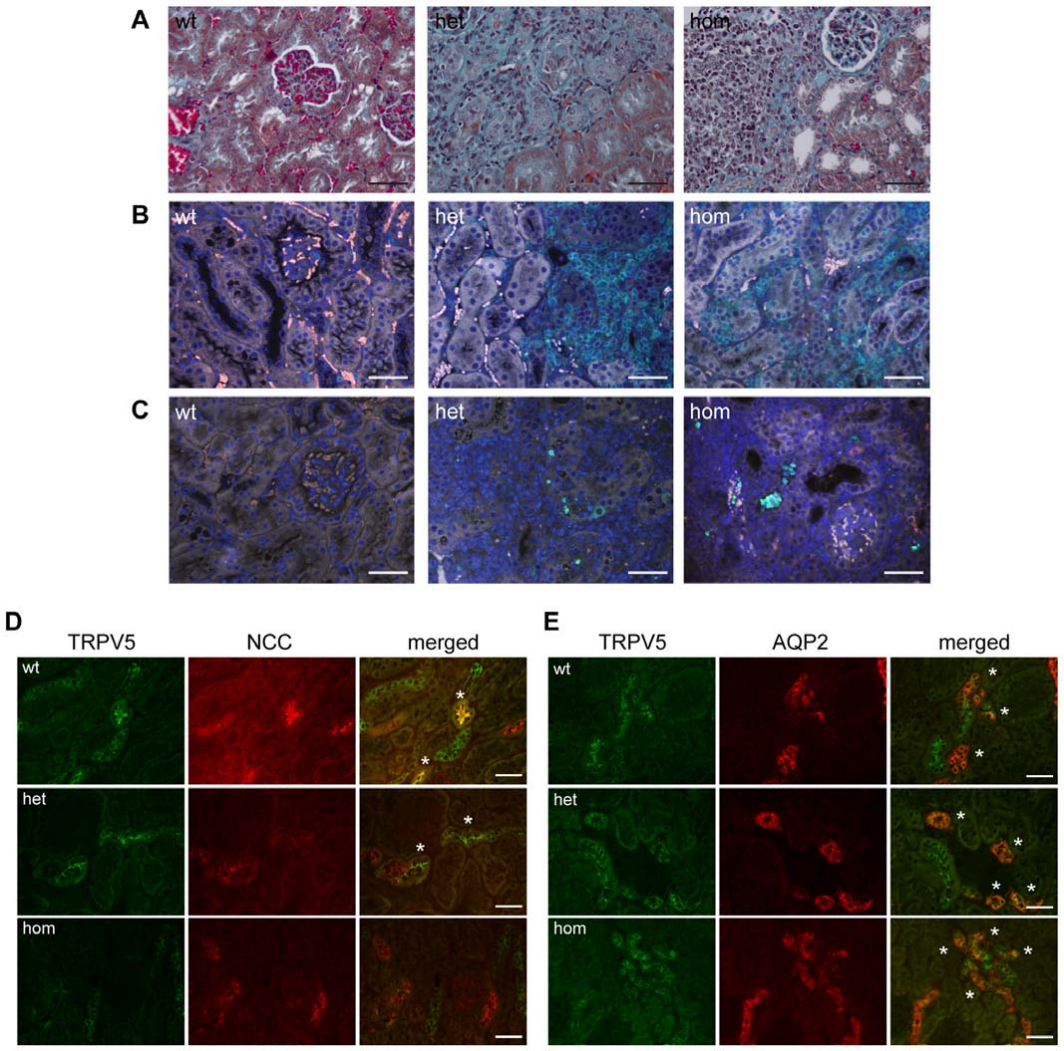
Histological and immunohistochemical assessment of kidneys from HCALC1 mice. Representative images in *Trpv5^+/+^* (wt), *Trpv5^682P/+^* (het) and *Trpv5^682P/682P^* (hom) mice of: (**A**) Masson's trichrome staining of renal cortex showing areas of interstitial fibrosis in *Trpv5^682P/+^* and *Trpv5^682P/682P^* mice (light blue), (**B**) anti-CD3-labelling (green) showing a large number of T-lymphocytes present in the interstitial regions of the *Trpv5^682P/+^* and *Trpv5^682P/682P^* mouse kidneys, (**C**) TUNEL-labelling (green) of the renal cortex showing the presence of tubular cell apoptosis in the *Trpv5^682P/+^* and *Trpv5^682P/682P^* mouse kidneys. Scale bar  =  50 µm. (**D**) Immunohistochemical images of kidney sections from wild-type (wt), *Trpv5* ^682P/+^ (het) and *Trpv5* ^682P/682P^ (hom) mice, co-stained for TRPV5 (green) and NCC (red). * denotes co-localisation. Scale bar  =  50 µm. (**E**) Kidney sections from wild-type (wt), *Trpv5* ^682P/+^ (het) and *Trpv5* ^682P/682P^ (hom) mice, co-stained for TRPV5 (green) and AQP2 (red). * denotes co-localisation. Scale bar  =  50 µm.

Despite the frequency of interstitial renal cortical calcification being similar in the HCALC1 and *Trpv5^+/+^* mice, ∼10% of *Trpv5* ^682P/+^and *Trpv5* ^682P/682P^ male mice had unilateral or bilateral smaller kidneys (Figure S2) associated with scarring, whereas 0% of *Trpv5* ^+/+^ males had such abnormalities. Histological examination revealed the presence of interstitial renal fibrosis, associated with inflammatory cell infiltrates, tubular dilatation, flattening of tubular epithelia and the presence of numerous cells and/or cell debris within the dilated lumen of some cortical tubules in kidneys of ∼10% of *Trpv5* ^682P/+^and *Trpv5* ^682P/682P^ male mice (Figure 3A). Immunohistochemical staining with antibodies against CD3, part of the T-cell receptor complex, confirmed an infiltration of T-cells within the interstitial regions of the renal cortex of affected kidneys from the *Trpv5* ^682P/+^and *Trpv5* ^682P/682P^ male mice, which was absent in *Trpv5* ^+/+^ kidneys (Figure 3B). In addition, TUNEL-staining showed apoptosis of renal tubular cells in the *Trpv5* ^682P/+^and *Trpv5* ^682P/682P^ male mice, not observed in *Trpv5* ^+/+^ kidneys (Figure 3C). These features are consistent with tubulointerstitial nephritis [27], [28].

Hypercalciuria may be associated with increased bone resorption leading to lower bone mineral density (BMD) and osteoporosis. We therefore assessed for bone abnormalities in the HCALC1 mice using dual-energy X-ray absorptiometry (DEXA), micro-computed tomography (microCT) scanning and histology. DEXA analysis of the femurs of 19–22 week-old *Trpv5* ^+/+^, *Trpv5* ^682P/+^and *Trpv5* ^682P/682P^ mice (n = 8–22/group) did not reveal any significant differences (in g/cm^2^: 0.075±0.001, 0.075±0.001, and 0.071±0.002 for *Trpv5* ^+/+^, *Trpv5* ^682P/+^and *Trpv5* ^682P/682P^ female mice, respectively; 0.071±0.001, 0.070±0.001, and 0.066±0.002 for *Trpv5* ^+/+^, *Trpv5* ^682P/+^and *Trpv5* ^682P/682P^ male mice, respectively) In addition, histological analysis of the femora from males and females did not reveal any morphological abnormalities in the *Trpv5* ^682P/+^ or *Trpv5* ^682P/682P^ mice when compared to *Trpv5* ^+/+^ mice (Figure S3). Furthermore, microCT scanning did not reveal any of the major abnormalities associated with osteoporosis, such as a reduction in trabecular bone volume [29], in the *Trpv5* ^682P/+^ and *Trpv5* ^682P/682P^ mice when compared to *Trpv5^+/+^* mice (Table S1); however, female *Trpv5* ^682P/682P^ mice were found to have a significantly elevated bone surface/volume ratio and reduced trabecular thickness which may be consistent with a deterioration in the microarchitecture of the bone.

### Effects of TRPV5-S682P mutation on TRPV5 and Calbindin-D_28K_ renal expression

**Figure 4:**
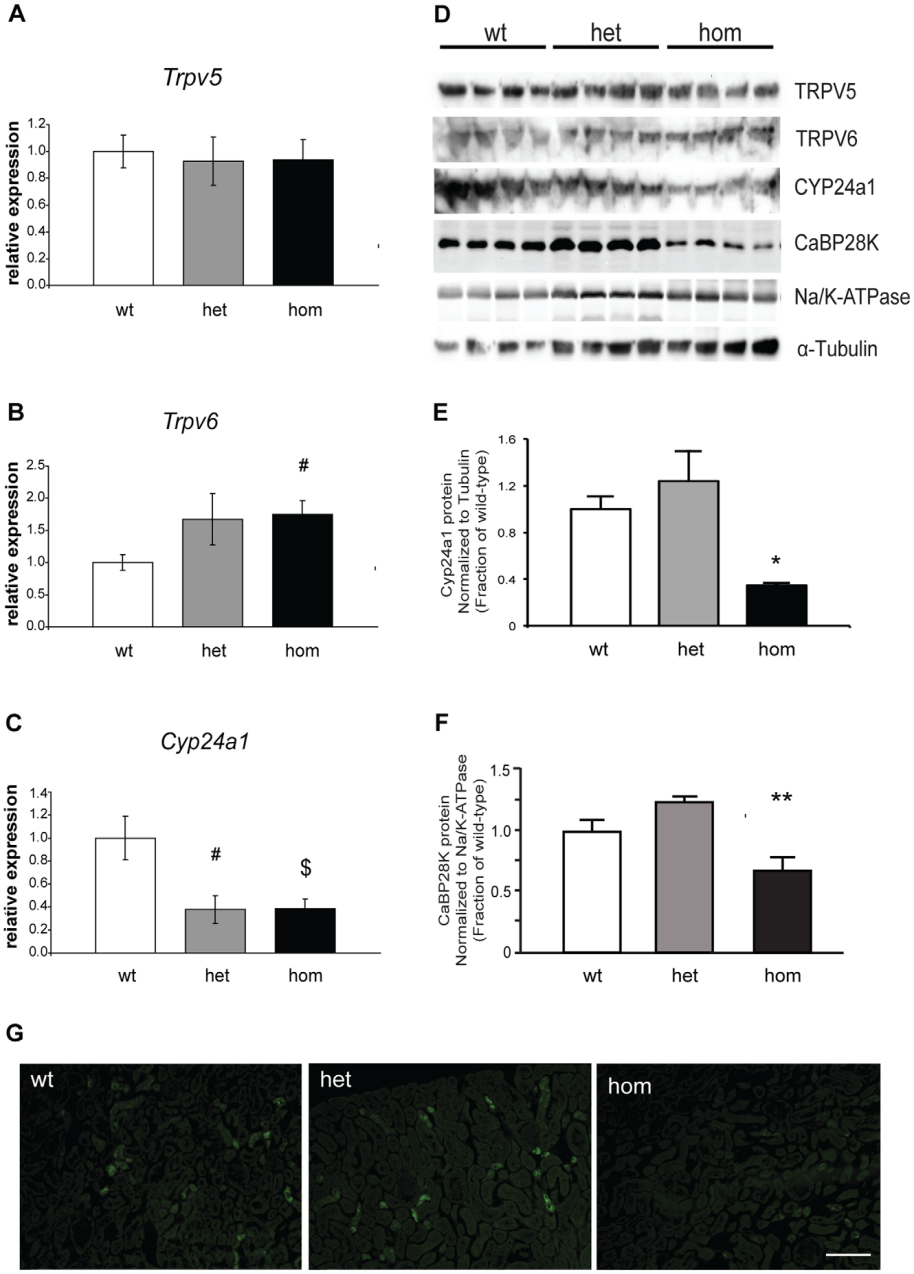
Assessment of Renal Expression of Calcium Regulatory Genes and Proteins. Renal expression of (**A**) *Trpv5*, (**B**) *Trpv6* and (**C**) *Cyp24a1* in wild-type (wt), *Trpv5* ^682P/+^ (het) and *Trpv5* ^682P/682P^ (hom) mice (n = 6/group) were assessed by quantitative real-time PCR. All data were normalised to levels of the housekeeping gene *Gapdh* and wild-type values are expressed as 1. Histogram data are presented as mean ± SEM. ^#^p<0.05, ^$^p<0.01, compared to wild-type mice. (**D**) Western blot analysis of TRPV5, TRPV6, CYP24a1 and calbindin-D_28K_ (CaBP28K) in kidneys of wild-type (wt), *Trpv5* ^682P/+^ (het) and *Trpv5* ^682P/682P^ (hom) mice. The α-1 subunit of the Na/K-ATPase and α-tubulin were used as housekeeping genes to normalize for equal loading. (**E**) Semi-quantitative densitometry analysis of CYP24a1. (**F**) Semi-quantitative analysis of CaBP28K. All histogram data are presented as means ± SEM. *p<0.05 and **p<0.02 compared to wild-type mice. (**G**) Immunohistochemical images of calbindin-D_28K_ (CaBP28K) stained kidney sections from wild-type (wt), *Trpv5* ^682P/+^ (het) and *Trpv5* ^682P/682P^ (hom) mice. Scale bar  =  200 µm.

The effects of the S682P mutation on TRPV5 renal expression, were assessed using kidney cryosections from *Trpv5^+/+^, Trpv5* ^682P/+^ and *Trpv5* ^682P/682P^ mice and anti-TRPV5 antibodies. Co-staining with antibodies against the thiazide-sensitive sodium/chloride co-transporter (NCC) or aquaporin-2 (AQP2) was performed to distinguish TRPV5-expression in the DCT and CNT, respectively, as NCC is expressed at the apical regions of DCT cells, with a decrease in expression towards the most distal part of the DCT segment, [24]_ENREF_18 whilst AQP2-expression commences at the CNT and extends throughout the collecting ducts [30], [31]. In *Trpv5^+/+^* mice, TRPV5-immunostaining was observed in the apical regions of the second half of the DCT (DCT2), and in the cytoplasmic regions of CNT cells (Figure 3D–E). By contrast, in *Trpv5* ^682P/682P^ mice, TRPV5-immunofluorescence was reduced, especially in NCC-positive tubular cells where TRPV5-staining was absent or appeared diffusely cytoplasmic (Figure 3D–E). Examination of TRPV5-NCC co-stained sections revealed that in *Trpv5* ^682P/+^ kidneys, TRPV5-expression appeared confined to the DCT2 distal portion where NCC-immunostaining was weakest (Figure 3D). The number of TRPV5-NCC co-positive cells in the kidneys of *Trpv5* ^682P/+^ and *Trpv5* ^682P/682P^ mice were significantly reduced (p<0.05) in comparison to *Trpv5* ^+/+^ kidneys (100±13%, 56±6%, and 27±5% for *Trpv5^+/+^, Trpv5* ^682P/+^ and *Trpv5* ^682P/682P^ mice, respectively, n = 5 mice/group, ≥3 different fields/kidney section). These differences in protein expression were not due to differences in transcription, as quantitative PCR analysis demonstrated that renal *Trpv5* mRNA levels in *Trpv5^+/+^, Trpv5* ^682P/+^ and *Trpv5* ^682P/682P^ mice were similar (Figure 4A). Thus, these findings are consistent with the mutant TRPV5 channel resulting in a probable intracellular trafficking defect. Interestingly, renal *Trpv6*-expression was significantly increased in *Trpv5* ^682P/682P^ mice compared to *Trpv5^+/+^* mice (Figure 4B), thereby suggesting a possible compensatory mechanism. Furthermore, expression of *Cyp24a1*, which encodes the 1,25-dihydroxyvitamin D_3_ 24-hydroxylase, the enzyme that inactivates 1,25-dihydroxyvitamin D_3_, was significantly decreased in *Trpv5* ^682P/+^ and *Trpv5* ^682P/682P^ mouse kidneys by qPCR (Figure 4C) and Western blot analysis (Figure 4D, E), consistent with the hypervitaminosis D observed in *Trpv5* ^682P/682P^ mice (Table 1). Semi-quantitative analysis of CYP24a1 and TRPV5 protein using whole kidney lysates confirmed the results obtained by qPCR, and revealed that CYP24a1 expression was decreased in *Trpv5* ^682P/+^ and *Trpv5* ^682P/682P^ mice (Figure 4E), and that TRPV5 expression was similar in wild-type, *Trpv5* ^682P/+^ and *Trpv5* ^682P/682P^ mice (data not shown). Similar semi-quantitative analysis of TRPV6 protein (data not shown) did not confirm the observed upregulation of mRNA levels (Figure 4B), although it should be noted that Western blot and densitometric analysis is less sensitive than quantitative PCR.

Expression of the intracellular vitamin D-regulated calcium-binding protein calbindin-D_28K_, which is co-expressed with TRPV5 in the DCT/CNT and is important for facilitating cytosolic calcium diffusion from the apical to the basolateral part of the epithelial cell, [32]_ENREF_25 was investigated. Semi-quantitative Western blot analysis revealed that calbindin-D_28K_-expression was significantly decreased in *Trpv5* ^682P/682P^ kidneys but not in *Trpv5* ^682P/+^ kidneys (100±6%, 125±14%, 52±3% for *Trpv5^+/+^, Trpv5* ^682P/+^ and *Trpv5* ^682P/682P^ mice, respectively, p<0.02) (Figure 4D, F), an observation confirmed by immunohistochemical analysis, (100±11%, 109±26%, 8±2% for *Trpv5* ^+/+^, *Trpv5* ^682P/+^ and *Trpv5* ^682P/682P^ mice, respectively, p<0.01) (Figure 4G).

## Discussion

Our studies have identified a novel mouse model, HCALC1, for autosomal dominant hypercalciuria harbouring a S682P mutation in TRPV5. Importantly, we have provided evidence that this mutation underlies the observed hypercalciuria as: *i)* the T to C substitution in codon 682 of mouse TRPV5 results in substitution of a highly conserved serine residue, making it unlikely to be a silent polymorphism (Figure 1); *ii)* the serine to proline change results in reduced basal [Ca^2+^]_i_ level in HEK293 cells expressing TRPV5-S682P, indicating a defect in the TRPV5-mediated calcium permeability of the cell (Figure 2); *iii)* expression of TRPV5 in *Trpv5* ^682P/+^ and *Trpv5* ^682P/682P^ kidneys was altered, particularly in the DCT2 (Figure 3D), consistent with a trafficking defect of the mutant TRPV5; and *iv)* renal calbindin-D_28K-_expression in *Trpv5* ^682P/682P^ mouse kidneys was reduced, further supporting a specific defect in TRPV5-mediated calcium reabsorption (Figure 4). Moreover, these findings reporting a role for the TRPV5-S682P mutation in the aetiology of the hypercalciuric phenotype are in agreement with the observations previously reported from mice that are null for Trpv5 (*Trpv5* ^-/-^) [25]. Thus, inactivation of TRPV5, in these null mice resulted in a decrease in renal calcium reabsorption, leading to severe urinary calcium loss, and normocalcaemia was maintained by a compensatory 1,25-dihydroxyvitamin D_3_–mediated increase in intestinal calcium absorption. Furthermore, in the *Trpv5^-/-^* mice the increased plasma 1,25-dihydroxyvitamin D_3_ concentrations were not associated with increased bone resorption because of TRPV5 inactivation in osteoclasts, and this situation is also observed in the *Trpv5* ^682P/+^ and *Trpv5* ^682P/682P^ mice (Table 1 and Table S1), thereby indicating that TRPV5 exerts its effects on extracellular calcium homeostasis principally by regulating renal calcium reabsorption. Interestingly, these patterns of calcium handling in the *Trpv5* ^682P/+^, *Trpv5* ^682P/682P^ and *Trpv5* ^-/-^ mice, are similar to those found in many patients with idiopathic hypercalciuria, who have renal hypercalciuria in association with elevated concentrations of 1,25-dihydroxyvitamin D_3_, but with normocalcaemia (or mild hypocalcaemia) and normal (or suppressed) plasma PTH concentrations [14], [21].

TRPV5 is a major protein involved in renal active calcium reabsorption, although to date, no TRPV5 mutations have been identified in patients with hypercalciuric kidney stone disease [33], [34]. However, only 29 unrelated patients have been studied, and this suggests the possibility that TRPV5 mutations may make a minor contribution and occur in <3% of patients with hypercalciuric renal stone disease. Indeed, it seems likely that in a highly heterogenous disorder such as hypercalciuric renal stone disease, that multiple genes may be involved with each giving rise to an autosomal inherited disorder and/or making a contribution to a polygenic trait, which is the likely situation for hypercalciuric renal stone disease [17]. Moreover, two TRPV5 SNPs, A563T and L712F, which have been reported to exhibit an increased calcium influx in *Xenopus* oocyte assays when compared with the reference TRPV5 may potentially explain the lower urine calcium excretion and reduced risk of kidney stones in African-Americans in whom these SNPs occur more frequently [35]._ENREF_28 This increase in TRPV5 calcium uptake in the A563T variant was also observed under experimental conditions that mimicked the compound heterozygous state, or in combination with other TRPV5 non-synonymous SNP variations [35]._ENREF_28 Our study, which further establishes the role of TRPV5 in hypercalciuria, indicates that mutational analysis of larger cohorts of hypercalciuric patients is warranted to assess if TRPV5 mutations may contribute to 3% (or less), of hypercalciuric renal stone disease. In addition, the HCALC1 mouse established by this study provides a pre-clinical model to evaluate treatments (e.g. diet or drugs) for hypercalciuria as well as facilitating studies of the physiological role of TRPV5 in renal calcium excretion.

HCALC1 mice are hypercalciuric, hyperphosphaturic, polyuric, polydypsic and have low urine pH. However, renal calcification in HCALC1 mice was not different from wild-type despite extreme calciuresis. These features are similar to the reported phenotype in *Trpv5* ^-/-^ mice [25]._ENREF_19 It has been postulated that polyuria and low urine pH reduce the risk of calcium phosphate precipitation in hypercalciuric mice [25], [34]. Studies in *Trpv5* ^-/-^ mice have shown that increased luminal calcium activates the apical calcium-sensing receptor in collecting duct cells leading to AQP2 downregulation and increased activity of the proton pump H^+^-ATPase, resulting in polyuria and increased acid secretion into the urine, respectively [34]._ENREF_29 Abolition of this compensatory urinary acidification in *Trpv5* ^-/-^ mice by genetic ablation of the H^+^-ATPase B1 subunit resulted in severe calcium phosphate precipitation in the renal medulla [34]._ENREF_29 Similar mechanisms may also contribute to reducing the risk of calcium precipitation in the presence of hypercalciuria in HCALC1 mice. The extent of hypercalciuria in *Trpv5^682P/+^* mice (∼10-fold above wild-type) was similar to that reported for *Trpv5^-/-^* mice (∼6-fold above wild-type)[25]_ENREF_19, and in *Trpv5^682P/682P^* mice, this was significantly higher than both *Trpv5^682P/+^* and *Trpv5^-/-^* (∼20-fold above wild-type), despite TRPV5-S682P causing only an ∼40% decrease in basal calcium influx in HEK293 cells (Table 1, Figure 2E–F). It could be expected that complete loss of TRPV5 in *Trpv5^-/-^* would cause a greater degree of hypercalciuria than the S682P mutation of TRPV5, which retains some calcium permeability. However, there was a decrease in TRPV5 protein expression specifically in the DCT (Figure 4E), suggesting that the S682P mutation may affect TRPV5 trafficking or regulation, for example by changing the conformation of the cytoplasmic C-terminal domain, which contains several protein-binding and regulatory motifs that regulate the subcellular localisation and trafficking of TRPV5 [36], [37], [38], [39]. Such a trafficking defect of mutant TRPV5 would not be detected by the *Xenopus* oocyte or HEK293 cells heterologous expression systems (Figure 2 and Figure S1) as these over-express the ion channel proteins, but may be detected by immunohistochemistry experiments of the renal tubules, as these utilise the native expression of proteins and are therefore more relevant to the physiological state. Indeed, the results of the immunohistochemistry studies (Figure 3D-3E) are consistent with the mutant TRPV5-S682P channel resulting in a trafficking defect, which would not be detected in the *Xenopus* oocyte or HEK293 cell heterologous expression systems. A decrease in apical TRPV5 expression specifically in the DCT in combination with the lower basal calcium flux of TRPV5-S682P, would reduce calcium reabsorption in the DCT further. Another way in which TRPV5 function is regulated is by phosphorylation of S654, likely by protein kinase C (PKC) as a result of apical CaSR stimulation which increases calcium flux through TRPV5. The S682P mutation could be hypothesised to cause a conformational change in the C-terminus that may render S654 unavailable to PKC, or may itself cause loss of a phosphorylation site, thus further decreasing the function of TRPV5-S682P. The further increase in hypercalciuria in *Trpv5^682P/682P^* mice compared to *Trpv5^682P/+^* mice is likely due to the active TRPV5 channel consisting of four TRPV5 monomers that form a central pore [40]_ENREF_34. Thus in *Trpv5^682P/+^* mice, 1 in 16 TRPV5 tetramers will consist of four wild-type TRPV5 monomers, whilst in *Trpv5^682P/682P^* mice, all TRPV5 channels will consist of four mutant TRPV5 monomers. An assessment of this possibility of a dominant-negative effect by the mutant TRPV5 was not feasible by *in vitro* experiments using the heterologous expression systems and co-transfection of wild-type and mutant TRPV5 constructs as the mutation did not affect channel properties when expressed in the *Xenopus* oocytes (Figure 2A–D) or HEK293 cells (Figure S1).

Expression of the calbindin-D_28K_ protein was also significantly reduced in *Trpv5* ^682P/682P^ mice. Such reductions have been observed in models with defective calcium influx via TRPV5, such as the *Trpv5* ^-/-^ and *Klotho^-/-^* mice [25], [41]. This reduction in calbindin-D_28K_-expression is likely due to the associated hypervitaminosis D in *Trpv5^682P/682P^* mice (Table 1) and this finding is similar to that reported in the *Trpv5^-/-^* mice [42]. The elevated circulating 1,25-dihydroxyvitamin D_3_ concentrations of the *Trpv5^682P/682P^* mice are the likely result of decreased degradation as renal *Cyp24a1-*expression was found to be significantly decreased (Figure 4E). This observation suggests that cellular calcium concentration is an important regulator of calbindin-D_28K_-expression, overriding the stimulatory effects of vitamin D. The reduction in calbindin-D_28K_-expression coupled with the probable mislocalisation of TRPV5-S682P in DCT tubules could result in reduced calcium reabsorption, and thus explain the hypercalciuria observed in *Trpv5^682P/682P^* and *Trpv5^682P/^* ^+^ mice. Furthermore, reduction in the interaction between TRPV5 and calbindin-D_28K_ in cells expressing Trpv5-S628P could explain the normal channel characteristics observed in TRPV5-S682P channels (Figure 2), as mutation of calbindin-D_28K_, leading to loss of interaction with TRPV5, has previously been demonstrated not to affect TRPV5 channel characteristics [32]_ENREF_25_ENREF_25_ENREF_25.

Histological analysis of the kidneys of HCALC1 mice demonstrated an increase in inflammatory infiltrates and tubular damage in ∼10% of male HCALC1 mice, consistent with interstitial nephritis. We hypothesise that this is a consequence of a combination of several factors within the HCALC1 mice. HCALC1 mice are polyuric, resulting in an increased hydrostatic pressure within tubules that could lead to cell damage. This cellular damage may lead to increased uric acid release from the cells [43] and increased inflammatory cell infiltration (Figure 3A–B). A consequence of cellular damage and the inflammatory response may be apoptosis of cells as demonstrated by TUNEL staining within the HCALC1 mouse kidneys (Figure 3C).

Our investigations for bone abnormalities in the Trpv5 mutant mice did not reveal any significant abnormalities other than a decrease in trabecular thickness and an increase in the bone surface/volume in female *Trpv5^682P/682P^* mice (Table S1). Our finding of a decrease in trabecular thickness in the female *Trpv5^682P/682P^* mice is in agreement with the reported observations in the *Trpv5^-/-^* mice [25]. However, male *Trpv5^-/-^* mice, unlike male *Trpv5^682P/682P^* mice, also had a decrease in trabecular thickness, and *Trpv5^-/-^* male and female mice, unlike *Trpv5^682P/682P^* male and female mice, also had a reduction in cortical bone thickness [25]. The basis for these differences between the *Trpv5^-/-^* [25] and *Trpv5^682P/682P^* mutant mice, may involve at least three possibilities, which include: differences in the backgrounds of the strains; the ages at which the mice were investigated; and the severity of the mutation. Differences in strain background have been reported to profoundly alter expression of mutant phenotypes [44], and it is important to note that the *Trpv5^682P/682P^* mutant mice were on a C57BL/6J.C3H background, whereas the *Trpv5^-/-^* mice were on a 129.B6 background [25]; thus it seems likely these differences in strain background may contribute to the observed differences in trabecular thickness between the *Trpv5^682P/682P^* and *Trpv5^-/-^* males. In addition, the *Trpv5^682P/682P^* mice were significantly older than the *Trpv5^-/-^* male mice (19 to 22 weeks versus 8 to 9 weeks of age) [25] at the time of the study; thus the greater maturity and longer duration of androgen exposure of the *Trpv5^682P/682P^* male mice may have ameliorated any reduction in trabecular and cortical bone thickness. Finally, it seems likely that TRPV5-682P represents a less severe mutation than the Trpv5 deletion of the knockout mice, as indicated by the lack of any significant effect of the TRPV5-682P mutant channel properties (Figure 2A–2D). The *in vivo* role of TRPV5 in bone metabolism is not fully understood and the availability of two mutant mouse models for TRPV5, with differences in bone phenotypes, will help such future investigations. TRPV5 is expressed in osteoclasts at the ruffled border and contributes to bone resorption but it is not expressed in osteoblasts; in contrast TRPV6 is expressed in both osteoblasts and osteoclasts but at very low levels, which are ∼1% of those in the intestine, and TRPV6 is not involved in osteoblast Ca^2+^ uptake [31], [45], [46]. *Trpv5^-/-^* mice have been reported to have increased numbers of osteoclasts, due to stimulation of osteoclast precursors by the high circulating 1,25(OH)_2_D concentrations, but have reduced bone reabsorption due to a lack of TRPV5 activity [25], [46]. However, the basis of the observed *in vivo* reduction in cortical bone mass in the *Trpv5^-/-^* mice remains to be elucidated and it has been proposed that TRPV5 may directly regulate osteoclast differentiation and/or RANKL-induced Ca^2+^ signaling [47]. Investigation of the *Trpv5^682P/682P^* and *Trpv5^-/-^* mouse models, which represent hypomorph and null models, respectively may help to further elucidate the *in vivo* roles of TRPV5 in skeletal biology.

In summary, HCALC1 represents the first mouse model reported to have dominant hypercalciuria due to a missense mutation in *Trpv5*. In contrast to the *Trpv5^-/-^* model for hypercalciuria, the presence of TRPV5 with a point mutation in HCALC1 mice may help elucidate roles for the TRPV5 C-terminus in the regulation of TRPV5 activity and trafficking, and the role of TRPV5 in renal mechanisms of calcium homeostasis and in hypercalciuria.

## Methods

### Ethics Statement

All animal studies were carried our using guidelines issued by the Medical Research Council in ‘Responsibility in the Use of Animals for Medical Research’ (July 1993) and Home Office Project License Numbers 30/2250 and 30/2752. Experiments were approved by the Medical Research Council Harwell ethics committee, and all efforts were made to minimize suffering.

### Experimental Animals

Studies were performed in accordance with guidelines issued under the UK Home Office Project licence. Animals were maintained in specific pathogen-free facilities, in individual ventilated cages and a 12-hour light-dark cycle, with free access to food and water. Mice were fed on Rat and Mouse No. 3 diet containing 1.15% calcium, 0.82% total phosphorus and 4088.68 units/kg of vitamin D (Special Diets Services, Wytham, Essex, UK).

### Generation of mutant mice

ENU-mutagenesis of C57BL/6J male mice was performed as previously described [48]._ENREF_37 F1 mice were obtained by crossing ENU-mutagenised C57BL/6J male mice with C3H/HeH (C3H) female mice. G2 mice for inheritance testing and mapping studies were derived by mating the founder male mouse with C3H female mice, or by *in vitro* fertilisation of C3H eggs using sperm from the founder male. Homozygous mutant mice (*Trpv5* ^682P/682P^) were generated by intercrossing heterozygous mutant (*Trpv5* ^682P/+^) male and female mice.

### Phenotype screen

Sixteen-week old F1 male mice were kept in metabolic cages (Techniplast, Kettering, UK) for 24-hours with free access to food and water [26]. Mice were weighed before and after, and food and water intake was monitored. 24-hour urine samples were collected in the presence of sodium azide and blood samples were collected from lateral tail vein or the internal jugular vein in lithium heparin Microvette tubes (Sarstedt, Leicester, UK) following terminal anaesthesia as previously described [26]. Urine and plasma chemistry were measured using an Olympus AU400 multi-channel analyser [26], [49]. Urine parameters were calculated as a ratio of sample creatinine, and plasma calcium was adjusted for plasma albumin concentration as described previously [26]._ENREF_20 Mice were weighed before and after, and food and water intake was monitored. 24-hour urine samples were collected in the presence of sodium azide and blood samples were collected from lateral tail vein or the internal jugular vein in lithium heparin Microvette tubes (Sarstedt, Leicester, UK) following terminal anaesthesia as previously described [26]._ENREF_20 Urine and plasma chemistry were measured using an Olympus AU400 semi-automated clinical chemistry analyser [26], [49]. Serum parathyroid hormone (PTH) concentration was measured using an ELISA specific for mouse intact PTH (Immutopics, San Clemente, CA, USA) as previously described [50]. Urine parameters were calculated as a ratio of sample creatinine, and plasma calcium was adjusted for plasma albumin concentration as described previously [26]._ENREF_20 Mice with a urine or plasma parameter that was 2SD above or below the population mean were retested at 24 weeks of age.

### Genetic mapping and DNA Sequence Analysis

DNA was isolated from ear or tail biopsies using the Gentra PureGene DNA isolation kit (QIAGEN, Crawley, UK). A genome wide scan was performed on 13 mice by Pyrosequencing on the PSQ HS 96A Instrument (QIAGEN), using a panel of ∼60 informative SNPs, distributed at 20–30cM intervals across 19 autosomes. Further mapping was carried out using more mice and additional informative SNPs across the candidate interval. The exons, corresponding intron-exon boundaries and promoters of mouse *Trpv5* and *Trpv6* genes [22], [23] were PCR-amplified using gene specific primers (sequences provided on request). DNA sequences were determined by semi-automated DNA sequencing and the DNA sequence abnormality confirmed by restriction enzyme digest of PCR products, using methods previously described [16],_ENREF_10[51]._ENREF_38_ENREF_40.

### Bone Analysis

Dissected formalin-fixed femora from 19–22 week-old *Trpv5^+/+^*, *Trpv5^682P/+^* and *Trpv5^682P/682P^* mice (n =  7–22 per group) were examined by DEXA and microCT. DEXA was carried out using a PIXImus X-ray densitometer (GE Healthcare, Little Chalfont, UK). The acquired images were processed using the PIXImus v2.1 software. MicroCT analysis was carried out using a Skyscan microCT scanner (model 1172a, Skyscan, Belgium) at 50 kV and 200 µA using a 0.5 aluminium filter and a detection pixel size of 4.3 µm^2^. Images were captured every 0.7° through 180° rotation of each bone. Scanned images were reconstructed using Skyscan NRecon software and analysed using the Skyscan CT analysis software. Trabecular bone was measured over a 1 mm^3^ volume, 0.2 mm from the growth plate. Trabecular bone volume as proportion of tissue volume (BV/TV,%), trabecular thickness (Tb.Th, mm) and trabecular number (Tb. N, mm^-1^) were assessed in this region using the CT analysis software [52]. Histological analysis was performed using 5 µm sections of femora that were stained with H&E [49]. Images were collected on a Nikon Eclipse E400 microscope, equipped with a Nikon DXM1200C digital camera.

### Kidney histology and immunohistochemistry

Dissected kidneys were halved, fixed in 10% neutral-buffered formalin overnight, and embedded in paraffin wax. Four- µm sections were prepared and stained with H&E, Masson's Trichrome, and von Kossa for the presence of renal calcification as described previously [49]._ENREF_38 TUNEL-staining for detection of apoptotic cells was performed using the ApopTag Fluorescein *In Situ* Apoptosis Detection kit (Millipore, Watford, UK) according to the manufacturer's instructions. The presence of T-lymphocytes was detected using rabbit anti-CD3 monoclonal antibodies (ab16669, Abcam, Cambridge, UK), followed by secondary detection using Alexa Fluor 488-conjugated donkey anti-rabbit (Molecular Probes, Invitrogen, Paisley, UK). Stained sections were mounted in mounting medium containing DAPI (Vector Labs, Peterborough, UK).

For TRPV5 immuno-detection, 8- µm kidney cryosections were processed for immunofluorescence labelling as previously described [53]._ENREF_41 Kidney cryosections were co-stained with rabbit anti-TRPV5 (ACC-035, Alomone Labs, Jerusalem, Israel) and goat anti-AQP2 (sc-9882, Santa Cruz, Insight Biotechnology, Wembley, UK) polyclonal antibodies, or with goat anti-TRPV5 (sc-23379, Santa Cruz) and rabbit anti-NCC polyclonal antibodies, followed by the appropriate Alexa Fluor 488- or 594-conjugated secondary antibodies (Molecular Probes). Images were collected on a Nikon Eclipse E400 microscope, equipped with a Nikon DXM1200C digital camera. For comparison of fluorescence intensity, kidney sections stained for each antibody were photographed under identical exposure conditions for all mice and NIS-Elements BR 3.0 software used to count the number of TRPV5-positive and TRPV5-NCC co-positive cells.

For calbindin-D_28K_ detection, kidneys were fixed in 2% (w/v) periodate-lysine-paraformaldehyde (PLP), followed by overnight incubation in 15% (w/v) sucrose. Seven- µm cryosections were prepared and stained with rabbit anti-calbindin-D_28K_ and images taken using a 10x objective on a Zeiss fluorescence microscope (Sliedrecht, The Netherlands) and a Nikon DMX1200 digital camera. Semi-quantitative determination of calbindin-D_28K_ protein expression was performed using Image J (image processing program, NIH, USA), similar to previous publications [54]._ENREF_42.

### Western blot analysis

For quantification of renal expression, of TRPV5, TRPV6, CYP24a1 and Calbindin-D_28K_ proteins, mouse total kidney lysates were prepared and analysed as described previously [55]._ENREF_43 Proteins were separated using SDS-PAGE and electro-transferred to polyvinylidene fluoride membranes (Immobilon-P, Millipore Corporation, Bedford, MA, USA). Blots were incubated overnight with rabbit anti-TRPV5, anti-TRPV6, anti-CYP24a1 (all from Santa Cruz, Insight Biotechnology, Wembley, UK), anti-calbindin-D_28K_ polyclonal antibodies or mouse anti-Na/K-ATPase α1-subunit monoclonal antibodies (generously provided by Professor Michael J. Caplan, Yale University School of Medicine, New Haven, CT, USA). Subsequently, the blots were incubated with Alexa Fluor 680-conjugated goat anti-rabbit (Molecular Probes, Invitrogen) and IRDye 800 CW conjugated goat anti-mouse (LI-COR Biosciences GmbH, Bad Homburg, Germany) secondary antibodies or with HRP-conjugated goat-anti-rabbit secondary antibodies (Biorad Laboratories, UK). Immunoreactive protein was detected using the Odyssey infrared detection system (Westburg, Leusden, The Netherlands) or visualized using Pierce ECL Western blotting substrate (Thermo Fisher Scientific) on a BioRad Chemidoc XRS+ system [56] and densitometric analysis performed using Image J.

### Cell Culture and transfection

Human embryonic kidney (HEK293) cells were grown in Dulbecco's modified Eagle's medium (DMEM, Bio Whittaker Europe, Vervier, Belgium) containing 10% (v/v) fetal calf serum (PAA, Linz, Austria), 13 mM NaHCO_3_, 2 mM L-glutamine, and 0.01 mg/ml ciproxin at 37 °C in a humidity controlled incubator with 5% CO_2_. Cells were transiently transfected with the appropriate plasmids using polyethyleneimine (PEI, Brunswig/PolySciences Inc) with a DNA:PEI ratio of 6∶1. After 24 h, transfected cells were used for live-cell imaging experiments.

### Electrophysiology

*Xenopus* oocytes were prepared as previously described [57]. Oocytes were injected with ∼0.8 ng wild-type or mutant *TRPV5* cRNA. The final injection volume was 50nl per oocyte. Isolated oocytes were used 2 days after injection. For each batch of oocytes, both wild-type and mutant mRNA were injected, to enable direct comparison of their effects. Whole-cell currents were recorded from intact oocytes using the two-electrode voltage-clamp method, filtered at 1 kHz and digitized at 4 kHz. Oocytes were constantly perfused at 20–22°C with a solution containing 2.5 mM KCl, 87.5 mM NaCl, 1 mM MgCl_2_, 1.8 mM CaCl_2_ and 5 HEPES (pH 7.4 with KOH). Whole-cell currents were recorded in response to 400 ms test pulses to various potentials (from –100 to +60 mV in 10 mV increments) from a holding potential of 0 mV. Current versus voltage relationships were constructed by measuring the current at the end of each pulse and plotting it against the test pulse potential. Data was plotted using Origin 7 (OriginLab, Northampton, MA, USA).

Recordings in HEK293 cells were performed as described in detail previously [58]. Briefly, cells were placed in an extracellular bath solution (150 mM NaCl, 6 mM CsCl, 10 mM glucose, 10 mM HEPES/NaOH, pH 7.4). Currents were determined in the tight seal whole-cell configuration using a patch clamp amplifier controlled by Patchmaster software (HEKA, Lambrecht, Germany). Cells were kept in nominal divalent free solution to prevent calcium overload. Patch pipettes had resistances between 1 and 4 mΩ after filling with standard pipette solution (20 mM CsCl, 100 mM Cs-aspartate, 1 mM MgCl_2_, 4 mM Na_2_ATP, 10 mM BAPTA, 10 mM HEPES/CsOH, pH 7.2.). Access resistances and capacitance were continuously monitored using the automatic capacitance compensation of the Patchmaster software. A linear voltage ramp protocol from -100 mV to +100 mV (within 450 ms) was applied every 2 s from a holding potential of 20 mV to measure current-voltage (I/V) relations. Ca^2+^ currents were measured for 2.5 s at −100 mV stepping from a holding potential of +70 mV. Current densities, expressed in units of membrane capacitance, were calculated from the current at −80 mV during the ramp protocols (by normalizing the current amplitude to the cell membrane capacitance). All experiments were performed at room temperature. The analysis and display of patch clamp data were performed using Igor Pro software (WaveMetrics, Lake Oswego, USA).

For whole-cell patch clamp measurements, nominal divalent free solution contained in mM: 150 NaCl, 6 CsCl, 10 Glucose and 10 HEPES/NaOH, pH 7.4. To measure Na^+^-current density, 50 µM EDTA was added to the nominal divalent free solution to chelate divalent cations. 10 mM CaCl_2_ to the nominal divalent free solution to measure Ca^2+^-current density. The standard intracellular (pipette) solution contained in mM: 100 Cs-aspartate, 20 CsCl, 1 MgCl_2_, 10 BAPTA, 4 Na_2_ATP, 10 HEPES/CsOH pH 7.2. To adjust the intracellular Ca^2+^ concentration, the appropriate amount of CaCl_2_ was added in the presence of 10 mM BAPTA, as determined by the CaBuf program (ftp://ftp.cc.kuleuven.ac.be/pub/droog-mans/cabuf.zip).

### Video imaging of [Ca^2+^]_i_ using Fura-2-AM

Functional studies were performed using pEGFP vectors containing full-length wild-type (wt) and mutant (S682P) mouse *Trpv5* cDNA. HEK293 cells were seeded on fibronectin-coated coverslips (Ø 25 mm) and transfected with the appropriate pCINeo/IRES-EGFP vector. After 24 hr, cells were loaded with 3 µM Fura-2-AM (Molecular Probes) and 0.01% (v/v) Pluronic F-129 (Molecular Probes) in DMEM medium at 37°C for 20 min. After loading, cells were PBS-washed and allowed to equilibrate at 37°C in HEPES-Tris buffer (in mM: 132.0 NaCl, 4.2 KCl, 1.4 CaCl_2_, 1.0 MgCl_2_, 5.5 D-glucose and 10 HEPES, titrated to pH 7.4 with Tris). For Ca^2+^ free conditions, a similar buffer composition was used in which Ca^2+^ was substituted with 2 mM EDTA. After Fura-2 loading, cells were placed in an incubation chamber and attached to the stage of an inverted microscope (Axiovert 200 M, Carl Zeiss, Jena, Germany). Extracellular Ca^2+^ was changed using a perfusion system and resulting changes in cytosolic Ca^2+^ levels were monitored with Fura-2 excited at 340 nm and 380 nm using a monochromator (Polychrome IV, TILL Photonics, Gräfelfing, Germany). Fluorescence emission light was directed by a 415DCLP dichroic mirror (Omega Optical Inc., Brattleboro, VT, USA) through a 510WB40 emission filter (Omega Optical Inc.) onto a CoolSNAP HQ monochrome CCD-camera (Roper Scientific, Vianen, the Netherlands). The integration time of the CCD-camera was set at 200msec with a sampling interval of 3 s. All hardware was controlled with Metafluor 6.0 software (Universal Imaging Corporation, Downingtown, PA, USA). Quantitative image analysis was performed with Metamorph 6.0 (Molecular Devices Corporation, Sunnyvale, CA, USA). For each wavelength, the mean fluorescence intensity was monitored in an intracellular region and, for purpose of background correction, an extracellular region of identical size. After background correction, the fluorescence emission ratio of 340 nm and 380 nm excitation was calculated to determine the intracellular Ca^2+^ concentration. All measurements were performed at room temperature. Numerical results were visualized using Origin Pro 7.5 (OriginLab Corp., Northampton, MA, USA).

### Quantitative PCR Analysis

RNA was extracted from whole mouse kidney using Trizol (Invitrogen Life Technologies) as per manufacturers instructions [59]. cDNA was prepared from 1 µg of RNA using the Quantitect Reverse Transcription Kit (Qiagen). qPCR reactions were carried out using the Rotorgene Sybr Green Kit (Qiagen) in six independent samples on a Rotorgene 5 (Qiagen) as described previously [60]. All qPCR test samples were normalized to levels of the reference gene *Gapdh*. Threshold cycle (C_T_) values were obtained from the start of the log phase on Rotorgene Q Series Software and C_T_ values analysed in Microsoft Excel 97-2010 using the Pfaffl method [61]. Data for each gene was normalised to *Gapdh* and wild-type values expressed as 1. Data for *Trpv5^682P/+^* and *Trpv5^682P/682P^* mice were expressed relative to wild-type mice.

### Statistical analysis

Statistical significance between groups was determined by pair-wise comparisons using a two-tailed unpaired Student's *t*-test. For comparisons of urine and plasma parameters between *Trpv5* ^+/+^, *Trpv5* ^682P/+^ and *Trpv5* ^682P/682P^ mice, Bonferroni's correction for multiple comparisons was used.

## Supporting Information

Figure S1 **Channel characteristics of wild-type and mutant TRPV5 in HEK293 cells.** (A) Whole-cell Na^+^ currents in TRPV5-WT and TRPV5-682P transfected HEK293 cells and (B) their respective mean current-voltage relationships (TRPV5-WT, n = 7, black; TRPV5-682P, n = 10, red). (C) Whole-cell Ca^2+^ currents in TRPV5-WT and TRPV5-682P transfected HEK293 cells and (D) their respective mean current-voltage relationships (TRPV5-WT, n = 7, black; TRPV5-682P, n = 10, red). (E) Ca^2+^-dependent inactivation is unaltered in the TRPV5-682P mutant. (F) Whole-cell Na^+^ currents in TRPV5-WT and TRPV5-682P transfected HEK293 cells in the presence or absence of 100 nM Ca^2+^ in the intracellular solution (n = 5–8 cells) and their respective mean current-voltage relationships.
(JPG)

Figure S2 **Macroscopic findings in kidneys from wild-type (WT) and TRPV5 mutant male mice.** Approximately 10% of *Trpv5^682P/+^* and *Trpv5^682P/682P^* male mice had unilateral or bilateral smaller kidneys. Kidneys from (A) wild-type male mouse and (B) *Trpv5^682P/+^* mutant male mouse are shown. *Trpv5^682P/682P^* male mice who had smaller kidneys (data not shown) were similar to those observed in *Trpv5^682P/+^* mice.
(JPG)

Figure S3 **Histology of femora from HCALC1 mice.** Representative haematoxylin and eosin (H&E) stained sections from femora of *Trpv5^+/+^* (wt), *Trpv5^682P/+^* (het) and *Trpv5^682P/682P^* (hom) mice are shown from males and females. Scale bar  =  50 µm. The femora from the *Trpv5^+/+^*, *Trpv5^682P/+^* and *Trpv5^682P/682P^* mice were similar.
(JPG)

Table S1 MicroCT analysis of femora from 19–22 week old *Trpv5^+/+^*, *Trpv5^682P/+^* and *Trpv5^682P/682P^* mice.
(DOCX)
