# Supplementary figures and images for: Autosomal Dominant Hypercalciuria in a Mouse Model Due to a Mutation of the Epithelial Calcium Channel, TRPV5
Source: PLoS One. 2013 Jan 30;8(1):e55412. doi: 10.1371/journal.pone.0055412 (PMC3559602; doi:10.1371/journal.pone.0055412)

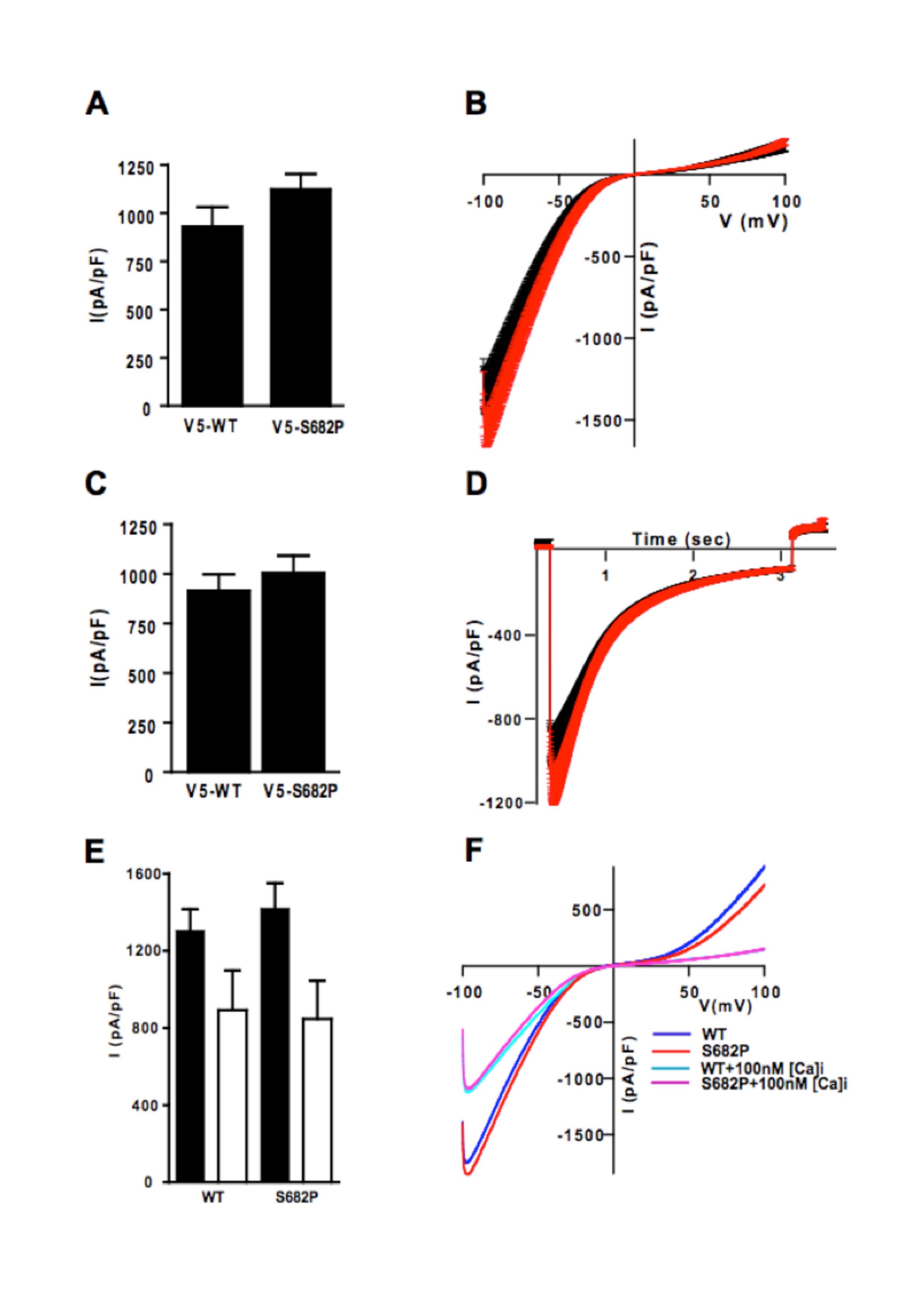

Supplement: Figure S1 — Channel characteristics of wild-type and mutant TRPV5 in HEK293 cells. (A) Whole-cell Na+ currents in TRPV5-WT and TRPV5-682P transfected HEK293 cells and (B) their respective mean current-voltage relationships (TRPV5-WT, n = 7, black; TRPV5-682P, n = 10, red). (C) Whole-cell Ca2+ currents in TRPV5-WT and TRPV5-682P transfected HEK293 cells and (D) their respective mean current-voltage relationships (TRPV5-WT, n = 7, black; TRPV5-682P, n = 10, red). (E) Ca2+-dependent inactivation is unaltered in the TRPV5-682P mutant. (F) Whole-cell Na+ currents in TRPV5-WT and TRPV5-682P transfected HEK293 cells in the presence or absence of 100 nM Ca2+ in the intracellular solution (n = 5–8 cells) and their respective mean current-voltage relationships. (JPG) [file pone.0055412.s001.jpg]

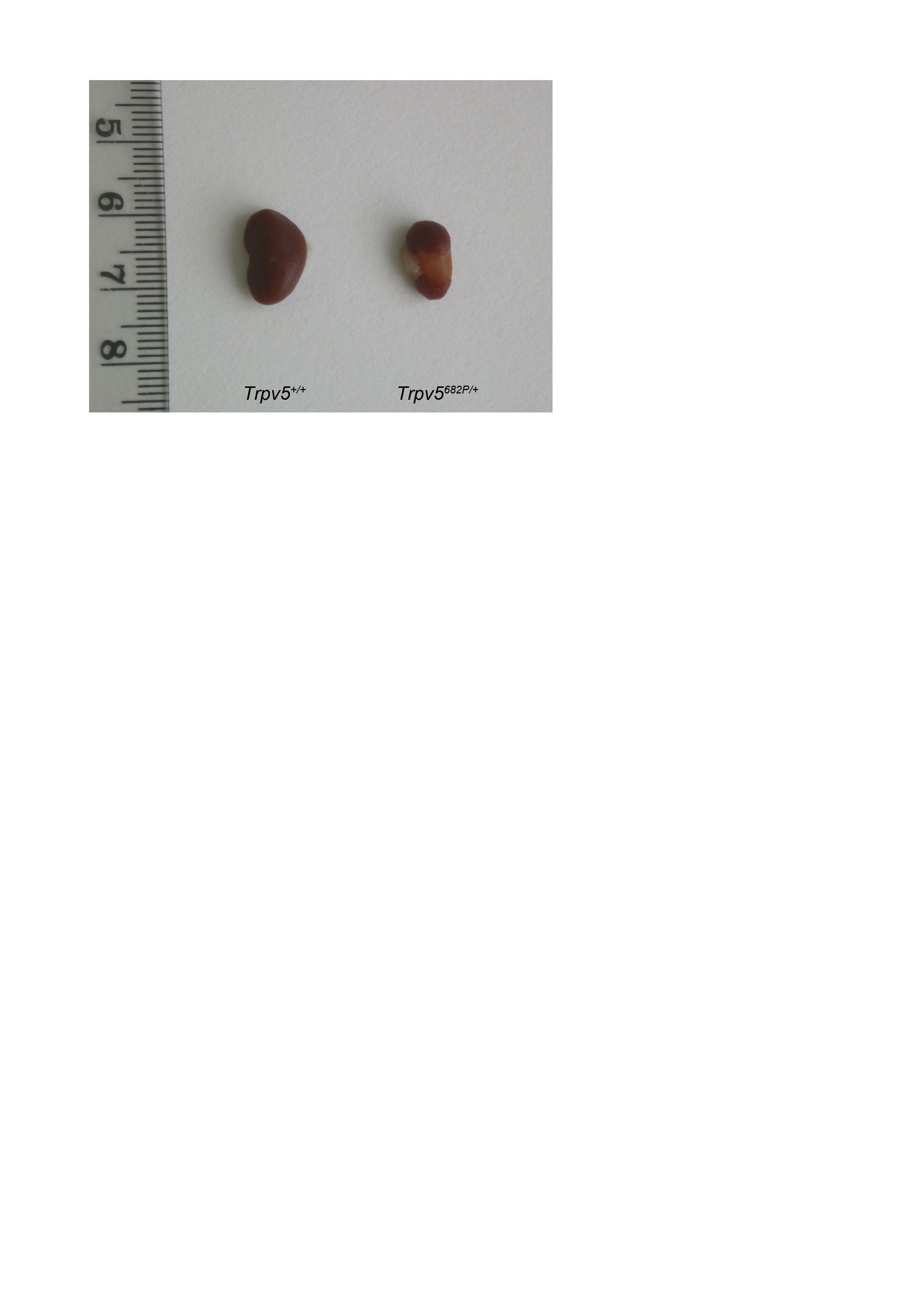

Supplement: Figure S2 — Macroscopic findings in kidneys from wild-type (WT) and TRPV5 mutant male mice. Approximately 10% of Trpv5682P/+ and Trpv5682P/682P male mice had unilateral or bilateral smaller kidneys. Kidneys from (A) wild-type male mouse and (B) Trpv5682P/+ mutant male mouse are shown. Trpv5682P/682P male mice who had smaller kidneys (data not shown) were similar to those observed in Trpv5682P/+ mice. (JPG) [file pone.0055412.s002.jpg]

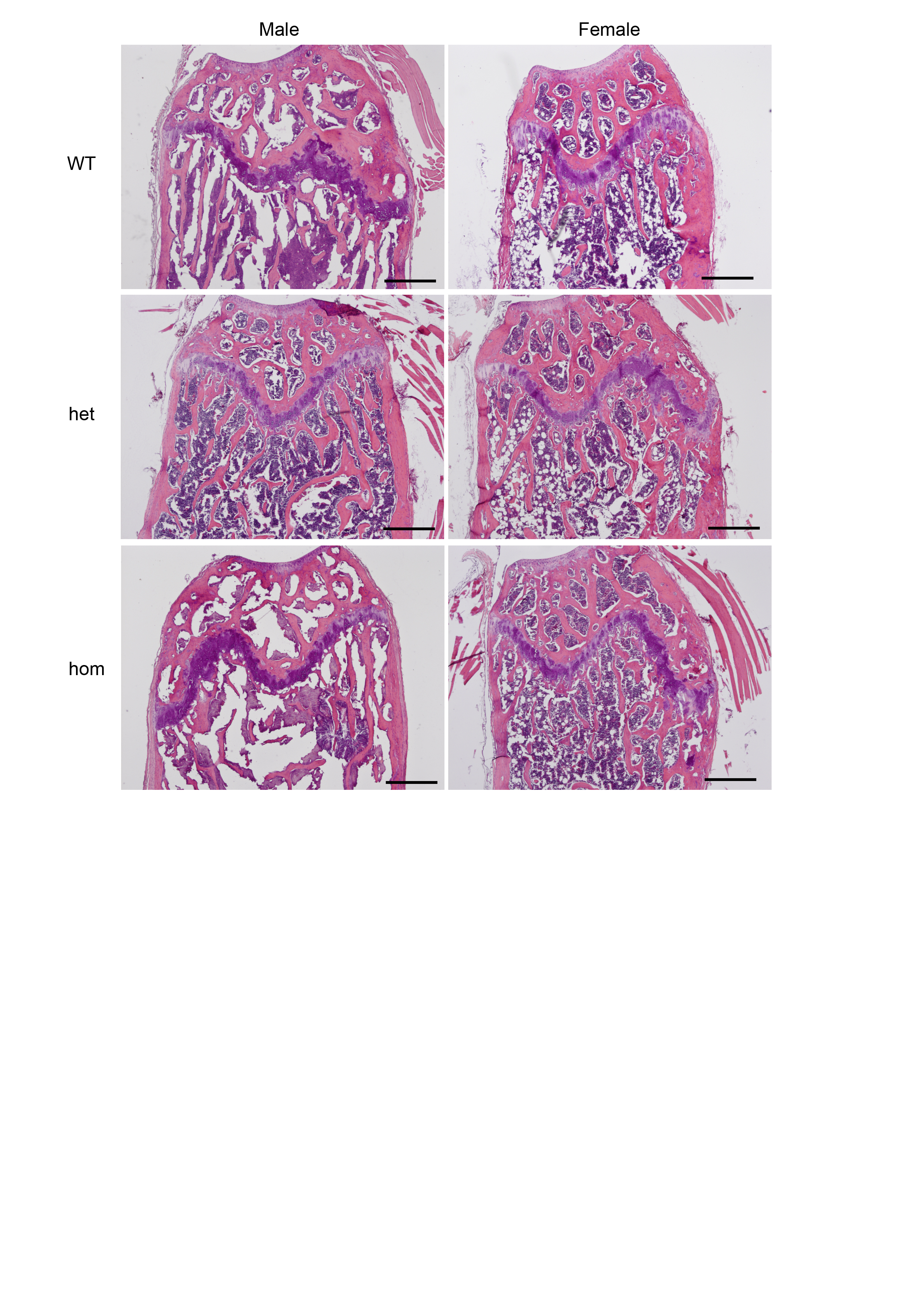

Supplement: Figure S3 — Histology of femora from HCALC1 mice. Representative haematoxylin and eosin (H&E) stained sections from femora of Trpv5+/+ (wt), Trpv5682P/+ (het) and Trpv5682P/682P (hom) mice are shown from males and females. Scale bar = 50 µm. The femora from the Trpv5+/+, Trpv5682P/+ and Trpv5682P/682P mice were similar. (JPG) [file pone.0055412.s003.jpg]
