# Supplementary material for: Autosomal Dominant Hypercalciuria in a Mouse Model Due to a Mutation of the Epithelial Calcium Channel, TRPV5
Source: PLoS One. 2013 Jan 30;8(1):e55412. doi: 10.1371/journal.pone.0055412 (PMC3559602; doi:10.1371/journal.pone.0055412)
Supplement: Table S1 — MicroCT analysis of femora from 19–22 week old Trpv5+/+, Trpv5682P/+ and Trpv5682P/682P mice. (DOCX) [file pone.0055412.s004.docx]

**Supplementary Table 1 MicroCT analysis of femora from 19-22 week old *Trpv5^+/+^*, *Trpv5^682P/+^* and *Trpv5^682P/682P^* mice**

|  |  | **Male** |  |  | **Female** |  |
| --- | --- | --- | --- | --- | --- | --- |
|  | **wt** | **het** | **hom** | **wt** | **het** | **hom** |
| **Tissue volume (mm^3^)** | 2.21±0.02 | 2.01±0.04 | 2.37±0.03 | 1.64±0.02 | 1.89±0.02 | 1.89±0.03 |
| **Bone volume (mm^3^)** | 0.43±0.01 | 0.32±0.01 | 0.46±0.03 | 0.41±0.02 | 0.37±0.02 | 0.30±0.01 |
| **Percent bone volume (%)** | 19.46±0.51 | 18.35±0.32 | 19.08±1.01 | 25.26±1.32 | 19.78±1.09 | 15.51±0.52 |
| **Tissue surface (mm^2^)** | 11.15±0.06 | 10.45±0.15 | 11.93±0.09 | 9.33±0.09 | 10.22±0.08 | 10.32±0.09 |
| **Bone surface (mm^2^)** | 29.35±0.73 | 23.70±0.98 | 30.23±1.03 | 21.15±0.66 | 23.42±0.54 | 21.65±0.79 |
| **Intersection surface (mm^2^)** | 1.65±0.08 | 1.45±0.05 | 1.68±0.09 | 1.73±0.10 | 2.18±0.09 | 1.19±0.05 |
| **Bone surface / volume ratio (mm^-1^)** | 68.78±0.89 | 74.38±0.74 | 70.83±2.12 | 53.89±1.34 | 62.68±1.18 | 73.83±0.58* |
| **Bone surface density (mm^-1^)** | 13.22±0.24 | 11.79±0.24 | 12.75±0.39 | 12.90±0.39 | 12.40±0.35 | 11.36±0.33 |
| **Trabecular thickness (mm)** | 0.05±0.01 | 0.05±0.01 | 0.06±0.01 | 0.07±0.01 | 0.06±0.01 | 0.05±0.01* |
| **Trabecular number (mm^-1^)** | 3.69±0.08 | 3.34±0.09 | 3.40±0.14 | 3.63±0.12 | 3.36±0.10 | 3.14±0.10 |
| **Trabecular pattern factor (mm^-1^)** | 8.78±0.74 | 9.02±0.60 | 2.45±1.57 | 4.65±0.58 | 4.93±0.74 | 3.29±0.42 |
| **Centroid (x) (mm)** | 1.00±0.02 | 0.77±0.03 | 0.95±0.02 | 0.90±0.02 | 0.76±0.02 | 0.96±0.02 |
| **Centroid (y) (mm)** | 1.00±0.03 | 1.16±0.03 | 1.20±0.03 | 0.89±0.03 | 1.15±0.02 | 1.04±0.03 |
| **Centroid (z) (mm)** | 2.56±0.05 | 2.64±0.02 | 2.29±0.08 | 2.37±0.08 | 2.62±0.08 | 1.94±0.09 |
| **Structure model index** | 1.34±0.04 | 1.20±0.03 | 1.14±0.05 | 1.38±0.04 | 1.27±0.05 | 1.00±0.03 |

Values are expressed as mean±SEM. * = p<0.02 with Bonferroni correction compared to *Trpv5^+/+^*. N=7 in each group.
